# Health Communication Film Implementation Strategy COVID-19-era: The Turning T.I.D.E. in HIV Multimedia Research

**DOI:** 10.33790/jswwp1100122

**Published:** 2024-09-13

**Authors:** Megan T. Ebor, Isabella M. Viducich, Madeline Y. Sutton

**Affiliations:** 1 Assistant Professor, School of Social Work, San Diego State University, United States.; 2 Department of Social Welfare, Luskin School of Public Affairs, University of California Los Angeles, United States.; 3 Department of Obstetrics and Gynecology, Morehouse School of Medicine, United States.

**Keywords:** COVID-19, Health Communication, Film, HIV, Implementation Methods

## Abstract

Among public health and social behavioral scientists there is an emergent interest in using film-based methodologies to promote health and wellness. During the novel coronavirus disease 2019 (COVID-19), this research method, as well as other forms of research, were thwarted. This article reports on our experience of implementing an online health communication film intervention under a research design initially intended for in-person communal viewing. We investigated if the film-based intervention could enhance the uptake of therapeutic modalities first learned through a brief five-week session for Black and Latine adults living with HIV. To address changes in research methods attributed to COVID-19, the T.I.D.E. implementation method includes four critical considerations: 1) *T-Tenacious* approach, increased level of persistence leveraging social and clinical supports, 2) *I- Immediate Needs*, connecting participants to needed community resources, 3) *D- Dissemination Plan* revamp and harness technology to share information, and 4) *E- Environmental Awareness* privacy protocol to strengthen trust by participants.

## Introduction

The onset of the novel coronavirus disease 2019 (COVID-19) disproportionately impacted racial/ethnic minorities who have long experienced health disparities, chronic diseases, and co-morbidities. As such, racial/ethnic minorities had increased vulnerability for COVID-19 related morbidity and mortality [1, 2]. Research also indicates that people living with HIV often have higher prevalence of risk factors for SARS-CoV-2 infection [1], e.g., hypertension, diabetes, cardiovascular disease, obesity, lung disease and smoking, [3] compared with their HIV-negative peers. Those co-morbidities also disproportionately impact Black and Latine populations.

As social distancing mandates and lockdowns were enforced at the onset of the pandemic in 2020–2021 (which was also an especially stressful time for Black and Brown communities in the U.S. due to excess COVID morbidity and mortality and the murder of George Floyd), community-based research initially ceased. Researchers and healthcare providers quickly pivoted away from traditional methods of practice toward implemention of COVID-19-era safe strategies in the United States, using methods including e-Mental health, telehealth, and digital interventions [4, 5]. While these adaptations were made swiftly, many of them were contingent upon access to technological resources that some racial/ethnic minorities and persons that are socioeconomically and/or geographically disadvantaged were less prone to access, e.g., home-based Internet service, hardware, software, [2, 4].

Multimedia interventions to enhance the impact and feasibility of STI/HIV prevention and care efforts have been gaining momentum over the past few decades. A meta-analysis describing mechanisms through which multimedia interventions may work to enhance delivery and uptake of STI/HIV interventions highlights several key approaches. This method has been used to promote condom use, increase HIV education and awareness for older adults via film-based programs, engage participants in interactive exercises to educate them about sexual risk behaviors and STIs through computer-based modalities, develop experiential programs using virtual reality to explore navigating safer sex negotiation, and implement web-based programs focused on skills-building and motivation [6–10]. These multimedia approaches demonstrate varying mediums of delivery some primarily relying on computer technology, while others are facilitator-led programs. These strategies and interventions were developed and implemented during a time when the complexities of a global pandemic were not a consideration. While racially disparate challenges in research, such as enrollment and retention, scarcity of resources, historical institutional untrustworthiness due to scientific bias and racism, and privacy concerns, existed prior to the pandemic, the COVID crisis greatly exacerbated these issues. Moreover, the large-scale structural challenges imposed by the digital divide, already a significant contributor to inequity, became even more pronounced.

Despite these overlapping risk factors (disparate impact of HIV, COVID-19, and medical co-morbidities), especially for people of color, there is a paucity of research reporting on the use of and lessons learned from community-informed multimedia research interventions during public health emergencies that may require shifts in community engagement. To inform this gap in research, we describe our methodology during the implementation phase of a multimedia health communication film intervention during the COVID-19 pandemic. We document experiential reflections on the enrollment and implementation process during an unprecedented public health crisis, especially among Black and Brown communities in the U.S., and suggest lessons learned that could strengthen engagement efforts during future public health emergencies.

## Methods/Procedures

The development of a film titled, “*TRY*,” (an acronym for, “Translating Research for You”) was approved by the Institutional Review Board at the University of California, Los Angeles (UCLA), IRB#20–000407 and was funded by the National Institutes of Health (NIH), National Heart, Lung, and Blood Institute (NHLBI) through a diversity supplement award (U01-HL142109–01). TRY (a 57-minute film, created by the first author) is a secondary level intervention designed to provide practical application exemplars through narrative storytelling. The film’s stories are told by three HIV-positive support group members who were affected by trauma. The goal was to encourage uptake of health affirming strategies taught via psycho-educational content of the parent study. The parent study at UCLA, from which participants were recruited, consisted of a novel blended, culturally congruent, evidence-informed care model entitled, “Healing our Hearts, Minds and Bodies” (HHMB; funded by the National Heart, Lung, and Blood Institute [NHLBI]) and was designed to address the intersecting issues of Black and Latine people living with HIV, patients’ trauma histories, barriers to care, and cardiovascular disease (CVD) risks [2, 11] all of which were conditions that were intensified at the onset and throughout the pandemic. Details of the development and theoretical underpinnings of the TRY film have been published elsewhere [2].

The T.I.D.E. implementation method is based on an acronym representing four critical considerations for enrollment and implementation of a film-based intervention during COVID-19. There is no particular order for which these considerations should be made. The acronym represents the following: 1) Tenacious approach to enrollment and retention– which involved leveraging social and clinical supports; 2) Immediate needs of the research participants connecting participants to needed community resources; 3) Dissemination plan modification– leveraging federal support to harness technology to continue community-based research implementation, and 4) Environmental awareness– ensuring protections and new protocols for confidentiality, privacy, and wellness of community participants. While film and arts-based methods for health and social behavioral interventions have become increasingly more of interest among researchers [7, 12–14], the impact, in part, has been effective because of the collective viewing experience, audience reach, and human connection [15–17]. The question which was addressed with our T.I.D.E. approach was: how can we mitigate structural challenges attributed to social and societal factors which may include the digital divide and medical co-morbid conditions) while implementing a film-based intervention during a public health crisis?

Prior to implementing the virtual film screenings, we tested the technology and implementation strategy among a small purposive sample of participants (N= 9) comprised of people previously enrolled in the parent study as part of a booster-session. Potential participants were called by either a member of the research team or clinician from the participants healthcare team. Once the participant agreed to attend the booster session, they were consented, enrolled, and scheduled to participate in a film viewing. A total of 10 separate film screenings were scheduled between October 2020 and April 2021. To test and refine the technology and virtual implementation strategy, participants were enrolled in a one session film screening spanning 2.0–2.5 hours. They watched the film remotely from thier homes or at a location with internet service. Post film viewing, each participant was provided a link to complete a 21-item questionnaire accompanied by a team member to help facilitate the completion of the online post-viewing questionnaire. Three items (17–19) were dedicated to the technical aspects of implementing the health communication film which is the focus of this paper. Sample items included the following: 1-“Did the “TRY” film have any technical problems or was it hard to understand?”. Responses to these questions were a yes/no variable and those that answered “yes” were provided a free-text box to provide details. We also asked, 2- “How did you watch TRY?, Responses included “tablet/iPad, computer, movie screen, other.” This question had a follow-up, “Was it easy to use?” and “Was the site we used (called “watch 2gether”) easy to use?” The third question asked was 3- “Did your environment cause any challenges to watching the film?” This series of questions had a yes/no variable and included a free-text box to provide specific details.

Experiential reflections and insights highlight the methods used to enroll and retain participants. The post-viewing questionnaire provided further understanding of the implementation strategy. Thus, the T.I.D.E. Implementation Method, detailed below, emerged through coupling our reflections/experiences as community-based researchers with the feedback provided by participants enrolled to test the implementation methods.

### Approaches/Implementation

Tenacious approach to enrollment and retention of participants. While difficulties in enrolling and retaining study participants in clinical research has been a longstanding concern particularly among racial/ethnic and socioeconomically disadvantaged populations for varying and justifiable reasons, e.g. mistrust, fear, and rigid research protocols, [18, 19] the onset of COVID-19 further emphasized complications in enrollment, retention, and implementation efforts in clinical trials and research more broadly [20]. Due to the inaccessibility of potential study participants resulting from stay-at-home orders in the State of California and the concurrent University-based cessation of in-person study implementation and recruitment [21], it was critically important to reevaluate traditional strategies while also keeping participants and study team mebers healthy and safe. Because our sample consisted of participants enrolled in the parent study who were living with HIV and were already in care at one of two participating agencies [11], we were able to tenaciously leverage social, clinical, and community members to support our research efforts. Therefore, we relied on the pre-pandemic service delivery systems and the clinical/support staff that provided services, care, and treatment for our study participants, in order to contact and enroll participants. For example, case managers placed in clinical settings, where our research participants received healthcare, played a vital role as liaisons to coordinate virtual attendance for film viewings. This is consistent with research indicating that recruitment strategies should involve engagement with clinic staff, research teams, and greater community engagement among researchers [19].

To promote retention, participants were provided with a wide range of options for scheduling their participation, including evening and weekend timeslots. Although the film screening involved a 1- session commitment spanning 2.0–2.5 hours, additional efforts were needed to ensure participant attendance on the scheduled day as life circumstances were increasingly volatile during this time. On occasion, provisions were made for participants to present via conference room for viewing when their built environment did not align with such programming (e.g. lack of privacy, small hoiusing quarters, etc.). Employing a coordinated effort to enroll and retain vulnerable populations in research during a public health crisis should involve flexible scheduling, intentional efforts to include clinicians and case managers as intermediaries, creative space accommodations, and added efforts to maintain contact and communication via text, email, or phone from a trusted research or clinical team member.

### Immediate Needs of Participants

Consistent with service delivery shifts in mental health care during the pandemic [4], the implementation of TRY also required a shift from skills teaching to prioritize assistance with resources to meet the basic needs of research participants. Ethical considerations for research during the pandemic included providing resources outside of the study focus. To accomplish this, we provided linkage to care outside of the scope of the study to prioritize a reciprocal benefit for both the community and science [22]. Therefore, we extended our programming to assist in meeting the expressed needs of study participants so not to overlook the vulnerability generated by circumstantial and structural barriers exacerbated by the pandemic. These considerations are especially important when conducting community-based research whereas one of the prominent outcomes is to attain balance between research and action that reciprocally adds value to both the community and scholarship [23, 24]. Consistent with research indicating the magnification of food insecurity among marginalized populations presenting a barrier to research due to disruptions of services during the pandemic [25, 26] there were times when study participants needed food, diapers, and/or assistance with housing— needs that were significantly greater than pre-pandemic times. Research teams should be prepared to identify and link current and potential study participants to resources that address immediate needs by leveraging community assets (e.g., faith-based and community organizations, temporary housing, meal delivery services, and medication/pharmacy delivery services). Once the immediate needs of the participants are met, researchers should continue to conduct programming while conducting consistent reflective oversight to identify and mitigate the vulnerabilities and challenges presented during research implementation.

### Dissemination Plan

The technological barriers, including limited home-based Internet service, disproportionately faced by racial/ethnic minorities and/or persons with lower socioeconomic status and educational levels, who also comprised the study population, necessitated a revised dissemination plan [2, 27]. To mitigate gaps in technology in the context of related social determinants of health, we addressed— built environment, education, and economic stability. Built environment identified as the lack of broadband Internet, limited access to community buildings, and housing insecurity [28], was partially addressed in the previous section (I-immediate needs), whereas study participants experiencing housing instability were connected to viable community resources through social services. Second, in consideration of economic stability, tablets and hotspots were attained and distributed as part of the larger parent study. Prior to and during the pandemic, these resources may have been financially out-of-reach or inconsistently accessible. Additionally, education was addressed by employing strategies to accommodate varying levels of digital literacy. For example, prior to distributing the equipment (e.g. tablets/hotspots), the research team set up the devices by fully charging batteries and downloading all applications (e.g., Zoom) that participants would utilize. Feedback from the post-film viewing questionnaire revealed that nearly 80 percent of our sample reported that they had no technical problems with the exception of two-responses indicating issues related to the sound.

The website “Watch2Gether” (https://w2g.tv/en/) was selected for virtual film screening due to its accessibility. Most notably, this platform did not require a log-in process, which was theorized as a barrier for participants that may be less technologically savvy. During all virtual film screenings, support staff were available via multiple avenues (phone, text, zoom chat) to troubleshoot any difficulties that arose and to reschedule participants if necessary. Participants were asked if the site, “watch2gether” was easy to use. Most respondents (~ 70 percent) reported that the site was easy to use. One respondent commented, “All I had to do was hit it and it popped right up.” The few responses indicating an issue stated, “ a little stumble at first but I got the hang of it.” Two participants reported that they watched it on another platform when they experienced challenges with “watch2gether.”

Research has shown that marginalized populations may also face economic sidelining that may lead to inconsistent access to technology [25, 26]. In this study, we recognize the privilege associated with an NIH affiliated parent grant which made accessing resources (tablets/hotspots) attainable— an advantage that many community-based research studies may not have. Other community-based telehealth interventions, including Rogers et al. [29], report success in utilizing participants’ existing technology paired with technological support and coaching. While our sample is not large enough to generalize our findings, it is noteworthy that five participants out of nine selected “other” when asked “How did you watch TRY?” and specified that they viewed the film via phone/mobile device. This finding supports the Rogers et al. [29] study which reports success in using participants’ existing technology. To this end, prospective participants and community-based interventions can benefit from resources such as the federal Lifeline Program which provides free cell phones and mobile internet data to eligible low-income Americans [30]. We also learned that a backup site for film viewing should be explored prior to fielding a film-based intervention to mitigate any issues related to accessing the platform used to view the film.

### Environmental Awareness

In addition to some study participants experiencing unstable housing during COVID, we were also cognizant of those residing in multigenerational, and/or constricted housing quarters that could potentially compromise participant privacy as well [31]. Thus, the pandemic-era shift to a safer virtual dissemination model that brought the intervention into participants’ homes, also required the adoption of protocols to protect the confidentiality of participants’ health and HIV status. For example, until an individual’s identity was verbally confirmed, researchers contacting current and potential participants via telephone introduced themselves as calling from the University by name rather than the specific HIV-associated lab conducting the study. Additionally, the research team remained sensitive to concerns around status disclosure that arose independent of living situation. Participants were also given the option to leave their web-cameras off and/or to employ a pseudonym as their screenname to maintain anonymity and privacy among other study participants while attending film viewings. Participants were asked, “Did your environment cause any challenges to watching the film?” If they answered “yes,” they were asked to specify. While a majority stated that there were no environmental challenges, one person stated, “It felt a bit lonely/quiet due to COVID—I don’t get to see as many people as before. But like the film said, we have to be strong.” While this response is not the focus of this paper, it does highlight the psychosocial impact of isolation and loneliness that people were facing at this time---both are critically important--- and may warrant consideration as potential barriers or facilitators to research implementation efforts during a pandemic.. Due to the environmental factors and personal preferences, telehealth interventions intended to reach these populations should adopt similar privacy protocols to avoid disclosure of their participants’ health information and/or HIV status.

## Conclusion/Discussion

We summarized our experiences of, reflections on, and lessons learned as we pivoted from in-person implementation to virtually disseminating a health communication film intervention during a public health emergency that required social distancing. This process involved a more critical and heightened approach to recruitment, retention, and implementation. As interest in multimedia approaches in social behavioral and public health research continues to rise, we must consider the implementation strategies that are best suited to reach the intended study population. While film-based intervention methods can be a viable tool for timely dissemination of accurate and effective messaging during a public health crisis, we must also contemplate the vulnerability generated by circumstantial and structural barriers, especially those experienced by disportionately affected Black and Brown persons. To mitigate structural challenges in implementing the current programming, we strategized and tested an online film-based intervention from which the T.I.D.E. methodology emerged.

As COVID required our research processes to be more iterative, we reflected and learned from participants and built upon heightened strategies for everyone’s safety and privacy. We strengthened digital options and created back-up plans to help remove barriers for our participants. We were proactive about providing social services that may have been high priority concerns during this very tumultuous public health and economic crisis time from 2020 through 2021.

Because our parent study allowed us to sample participants who self-identified as Black [32] and/or Latine, we were also successful in engaging with them by being mindful of the many structural and social challenges that were at the forefront in the U.S. during our 2020–2021 recruitment window [27]. Shifting approaches while also being purposeful about any social barriers and/or needs [33], and respectful about responding to questions or concerns about study intentions and goals, helped to facilitate our ability to collect data [34]. In addition, the free text options that were embedded within our questions allowed for sharing of feedback that was helpful for refining our processes and informing future strategies. The use of those qualitative research methods will allow researchers to delve more deeply into participants’ experiences, perspectives, and opinions in future analyses. By using open-ended questions and probing techniques, researchers can uncover rich and detailed information beyond mere surface-level responses. This type of “culture-centered approach” which involves being very intentional about listening, has been described [35] and can be insturumental in mobilizing communities to transform structural determinants of health.

In conclusion, we carefully constructed these four critical considerations to comprise the T.I.D.E. Implementation Method. This method emerged through our experiences of, reflections on, and lessons learned from pivoting from in-person implementation to virtually disseminating a health communication film intervention during COVID-19, and it allowed us to maintain, and in some cases, build new bridges to community members who participated in our study. These bridges are vital to building and maintaining trust with communities during regular interactions, and even more crucial during public health emergencies, especially with disproportionately affected people of color. This implementation method provides a good framework that can be considered during research studies that take place during pandemics and other emergencies.
